# A *var2 *leaf variegation suppressor locus, *SUPPRESSOR OF VARIEGATION3*, encodes a putative chloroplast translation elongation factor that is important for chloroplast development in the cold

**DOI:** 10.1186/1471-2229-10-287

**Published:** 2010-12-28

**Authors:** Xiayan Liu, Steve R Rodermel, Fei Yu

**Affiliations:** 1 College of Life Sciences, Northwest A&F University, Yangling, Shaanxi 712100, People's Republic of China; 2 Department of Genetics, Development and Cell Biology, Iowa State University, Ames, IA 50011, USA

## Abstract

**Background:**

The Arabidopsis *var2 *mutant displays a unique green and white/yellow leaf variegation phenotype and lacks VAR2, a chloroplast FtsH metalloprotease. We are characterizing second-site *var2 *genetic suppressors as means to better understand VAR2 function and to study the regulation of chloroplast biogenesis.

**Results:**

In this report, we show that the suppression of *var2 *variegation in suppressor line *TAG-11 *is due to the disruption of the *SUPPRESSOR OF VARIEGATION3 *(*SVR3*) gene, encoding a putative TypA-like translation elongation factor. SVR3 is targeted to the chloroplast and *svr3 *single mutants have uniformly pale green leaves at 22°C. Consistent with this phenotype, most chloroplast proteins and rRNA species in *svr3 *have close to normal accumulation profiles, with the notable exception of the Photosystem II reaction center D1 protein, which is present at greatly reduced levels. When *svr3 *is challenged with chilling temperature (8°C), it develops a pronounced chlorosis that is accompanied by abnormal chloroplast rRNA processing and chloroplast protein accumulation. Double mutant analysis indicates a possible synergistic interaction between *svr3 *and *svr7*, which is defective in a chloroplast pentatricopeptide repeat (PPR) protein.

**Conclusions:**

Our findings, on one hand, reinforce the strong genetic link between VAR2 and chloroplast translation, and on the other hand, point to a critical role of SVR3, and possibly some aspects of chloroplast translation, in the response of plants to chilling stress.

## Background

The photosynthetic apparatus of photosynthetic eukaryotic cells is the product of two genetic systems -- the nucleus-cytoplasm and the plastid. Nuclear-encoded chloroplast proteins usually have an N-terminal targeting sequence and are translated on cytoplasmic 80 S ribosomes as precursors; import into the organelle is accompanied by removal of the "transit" peptide to generate the mature protein (reviewed in [1]). The chloroplast genome, on the other hand, has many prokaryotic-like features - a remnant of the endosymbiotic origin of these organelles [2]. Chloroplast DNA-encoded proteins are translated on prokaryote-like 70 S ribosomes, usually in their mature forms, and assemble with nuclear-encoded counterparts to form a given multisubunit complex. The coordination and integration of the expression of nuclear and plastid genes involve both anterograde (nucleus-to-plastid) and retrograde (plastid-to-nucleus) regulatory signals that are elicited in response to endogenous cues, such as developmental signals, and exogenous cues, such as light [3-5].

Variegation mutants are ideal models for studying the mechanisms of chloroplast biogenesis. The Arabidopsis *variegation2 *(*var2*) mutant displays green and white/yellow patches in normally green organs. The green sectors contain morphologically normal chloroplasts while the white sectors contain abnormal plastids that lack chlorophyll and contain underdeveloped lamellar structures [6,7]. The variegation phenotype of *var2 *is a recessive trait and is caused by the loss of a nuclear gene product for an FtsH ATP-dependent metalloprotease that is targeted to chloroplast thylakoid membranes [7,8].

The function of FtsH-like proteases is best understood in *Escherichia coli *and yeast mitochondria where they play a central role in protein quality control and cellular homeostasis [9,10]. FtsH is thought to play similar roles in photosynthetic organisms, inasmuch as it is involved in turnover of damaged or unassembled proteins, including the photosystem II (PSII) reaction center D1 protein [11-21], the cytochrome b_6_f Rieske FeS protein [22], light harvesting complex II [23], and in cyanobacteria, unassembled PSII subunits [24]. FtsH proteins have also been implicated in membrane fusion and/or translocation events [25], the N-gene mediated hypersensitive response to pathogen attack [26], heat stress tolerance [27], and light signal transduction [28].

If VAR2 is required for chloroplast biogenesis, as evident by the formation of white sectors in *var2*, an intriguing question is how some cells of the mutant are able to bypass the requirement for VAR2 and form functional chloroplasts, despite having a *var2 *genetic background. A *threshold *model has been proposed to explain the mechanism of variegation in *var2 *[29]. This model is based on the observation that leaf cells of *var2 *are heteroplastidic, i.e. each of the many plastids in an individual cell acts in autonomous manner [6], and assumes that there is a fluctuating level of FtsH activity required for chloroplast function that reflects different micro-physiological conditions of individual developing plastids. In wild-type and the green sectors of *var2*, it is hypothesized that above-threshold levels of FtsH activity are present, and that these are sufficient for normal chloroplast development. Below-threshold activities, on the other hand, are not sufficient for chloroplast biogenesis and condition the formation of non-pigmented plastids. Our working hypothesis is that the green sectors of *var2 *have compensating factors/activities that either promote FtsH levels/activities or lower the FtsH threshold needed for chloroplast biogenesis. For example, the VAR2 homolog AtFtsH8 is a compensating factor [29].

To further dissect VAR2 function and to identify the factors/activities that enable normal chloroplast biogenesis in the absence of VAR2, we and others have carried out genetic screens for second-site *var2 *suppressors [30-32]. To date, a handful of suppressor mutants have been characterized at the molecular level (reviewed in [33]). Surprisingly, a majority of these have defects in the linked processes of chloroplast rRNA processing and chloroplast translation [31,32,34]. This argues for a linkage between VAR2 and these processes. It is also worth noting that the various suppressor lines have distinct accumulation patterns of chloroplast 23 S rRNA, suggesting that rRNA processing defects may not be a secondary effect of perturbed chloroplast function, but rather that they are a consequence of disruption of specific regulatory steps governing chloroplast rRNA processing [34].

In this study, we report the cloning and characterization of a *var2 *suppressor line designated *TAG-11*. We show that suppression of *var2 *in this line is caused by disruption of *SVR3*, a gene that encodes a chloroplast homolog of the *E. coli *TypA translation elongation factor. TypA is a member of the translation elongation factor superfamily of GTPases [35]. We show that *svr3 *single mutants and the *TAG-11 *double mutants (*svr3 var2*) have minor chloroplast rRNA processing defects and a moderate reduction of chloroplast protein accumulation at 22°C, with the exception of a sharp reduction in the level of photosystem II D1 protein. Interestingly, the *svr3 *single mutant has a chilling sensitive phenotype: at 22°C, it is pale green; while at 8°C it is chlorotic and has greatly reduced amounts of chlorophyll, aberrant chloroplast rRNA accumulation and processing, and abnormal chloroplast protein accumulation. Our findings suggest that SVR3 is involved in proper chloroplast rRNA processing and/or translation at low temperature. Taken together, the data presented here strengthen the link between VAR2 function and chloroplast translation. Furthermore, the chilling sensitive phenotype of *svr3 *provides more evidence that higher plant chloroplasts are intimately involved in the response of plants to chilling stress.

## Results

### Phenotype of a *var2 *suppressor line, *TAG-11*

**Figure 1:**
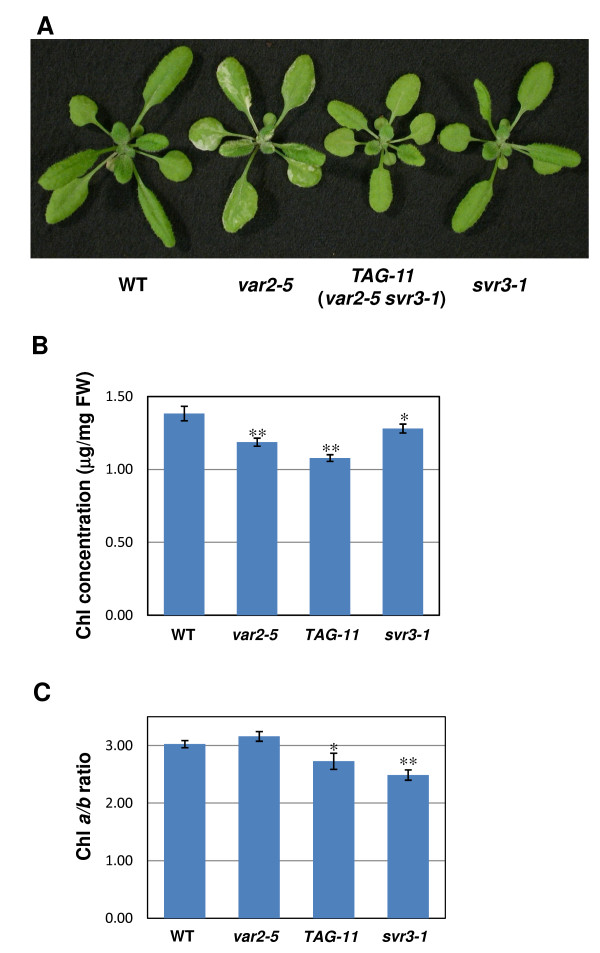
**Phenotypes of wild-type, *var2-5*, *TAG-11 *and *svr3-1 *grown at 22°C**. **(A) **Representative three-week old wild-type, *var2-5*, *TAG-11 *(*var2-5 svr3-1*) and *svr3-1 *single mutant plants. **(B) **Chlorophyll contents and **(C) **Chlorophyll *a/b *ratios in leaves from two-week-old wild-type, *var2-5*, *TAG-11 *(*var2-5 svr3-1*) and *svr3-1*. Error Bar represents the mean ± S.D. of three different samples and each sample consists of two seedlings (Chl: chlorophyll; **: p < 0.01; *: p < 0.05).

We have previously identified *var2 *suppressors via ethyl methanesulfonate (EMS) mutagenesis [30] and T-DNA activation tagging [32]. In this report, we describe a T-DNA-tagged *var2 *suppressor designated *TAG-11 *(Figure 1A). Analyses of F2 and F3 progeny from a cross between *TAG-11 *(generated in *var2-5 *background) and *var2-5 *indicated that the suppression phenotype in *TAG-11 *is due to a recessive mutation that co-segregates with a complex T-DNA insertion pattern at a single locus (Additional file 1, Figure S1). We named this locus *SUPPRESSOR OF VARIEGATION3 *(*SVR3*), and the allele in *TAG-11 *was designated *svr3-1*. To isolate *svr3-1 *single mutants, *TAG-11 *(*var2-5 svr3-1*) was backcrossed to wild-type Arabidopsis and the genotype of the *VAR2 *locus in the F2 progeny of the backcross was determined using derived cleaved amplified polymorphic sequence (dCAPs) primers [30,36]. Figure 1A shows that *TAG-11 *is smaller than wild-type and has pale green leaves due to significantly less chlorophyll than normal (Figure 1B). *TAG-11 *is also slightly variegated at later developmental stages. On the other hand, most of the phenotypes of *svr3-1 *are intermediate between those of *TAG-11 *and wild-type, including size, extent of variegation and chlorophyll content (Figure 1A-B). The exception is chlorophyll *a/b *ratios (Figure 1C), which are lower in *svr3-1 *than in the other lines. These observations are in contrast to other reported *var2 *suppressor lines, in which the *svr *single mutants and the suppressor lines have very similar phenotypes and the suppressor lines do not display visible variegation [30,32]. This suggests that the genetic interaction between *var2 *and *svr3 *is more complex than the epistatic relationships we have observed before.

### Identification of *SVR3*

**Figure 2:**
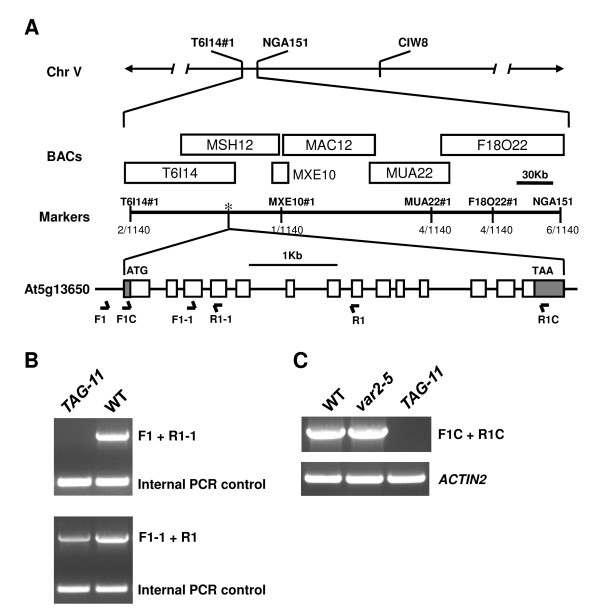
**Cloning of *SVR3***. **(A) **Procedure of map-based cloning of *SVR3 *is described in Methods. Markers used in fine mapping are listed in Additional file 1, Table S1. A total of 570 F2 plants (1140 chromosomes) were examined, and the number of recombinants is shown under each marker. The position of *SVR3 *(At5g13650) is indicated by the asterisk. In the gene model, boxes represent exons while solid lines represent introns. Shaded parts represent the 5' and 3' untranslated regions (UTRs). **(B) **and **(C) **Verification of the identity of *SVR3 *using PCR **(B) **and RT-PCR **(C)**. Primers used for PCR and RT-PCR are indicated by arrows in gene model in **(A)**.

The suppression of *var2-5 *leaf variegation in *TAG-11 *is linked with T-DNA insertion events, suggesting that the suppressor phenotype is likely caused by T-DNA insertions (Additional file 1, Figure S1). But due to the complexity of these events, plasmid rescue attempts were not successful in cloning *SVR3 *(Additional file 1, Figure S1). As an alternative approach, we used positional cloning to delimit the *SVR3 *locus to a ~123 kb interval on chromosome 5 using a series of molecular markers we designed using the Cereon genomics Indel and SNP databases (Figure 2A; [37]; all unpublished primers used in this report are listed in Additional file 1, Table S1). We reasoned that mutations that can cause suppression of *var2 *likely affect nuclear genes encoding chloroplast proteins. Six such genes reside in the ~123 kb interval. Because the mutation in *TAG-11 *is probably a complex T-DNA insertion, PCR using primers flanking wild-type genomic fragments containing the T-DNA insertion should fail to amplify wild-type sized fragments. Using this method we determined that At5g13650 is the gene bearing the mutation: as illustrated in Figures 2A and 2B, primers F1 and R1-1 failed to amplify a wild-type sized fragment of this gene from the mutant genomic DNA. The other five genes, by contrast, gave rise to wild-type sized fragments using other sets of primers to amplify *TAG-11 *genomic DNA. We further found that primers F1-1 and R1 amplified the same wild-type sized fragments with either *TAG-11 *or wild-type genomic DNA (Figure 2B), suggesting that the T-DNA insertion in At5g13650 likely resides between primers F1 and F1-1. Figure 2C shows that transcripts bearing the entire predicted coding region of At5g13650 are not detectable in *TAG-11 *by RT-PCR, suggesting that *svr3-1 *is a molecular null allele and offering further confirmation that At5g13650 is the suppressor gene. Although our data indicate that At5g13650 is disrupted by T-DNA insertion in *TAG-11*, we cannot completely rule out the possibility that the complex T-DNA insertion pattern in *TAG-11 *is a result of several individual insertion events at closely linked loci.

### Identification of *svr3-2*, a second allele of *svr3*

**Figure 3:**
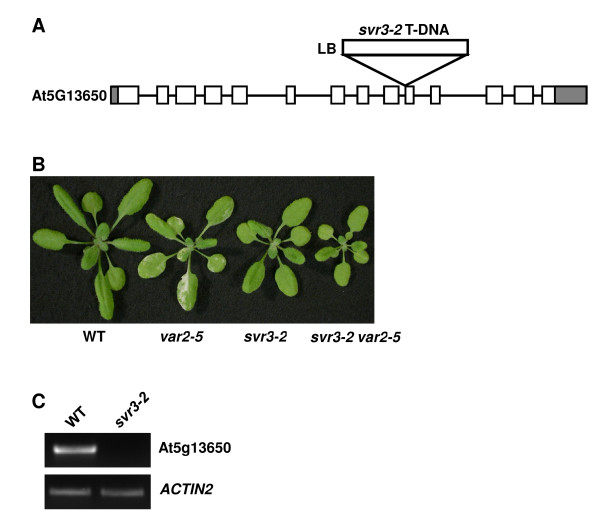
**Identification of *svr3-2***. **(A) **T-DNA insertion site in *svr3-2 *(SAIL_170_B11, CS871763). **(B) **Phenotypes of representative three-week-old wild-type, *var2-5*, *svr3-2 *and the *svr3-2 var2-5 *double mutant grown at 22°C. **(C) **Semi-quantitative RT-PCR analysis of At5g13650 expression in wild-type and *svr3-2*. Primers (13650F2 and 13650R3) used to detect At5g13650 transcripts are listed in Additional file 1, Table S1. *ACTIN2 *expression is shown as a control.

To verify that At5g13650 is the suppressor gene in *TAG-11*, we searched for a second mutant allele from publicly available collections of T-DNA insertion mutants http://signal.salk.edu/cgi-bin/tdnaexpress. One line (SAIL_170_B11; TAIL number CS871763) was reported to have a T-DNA insertion in the 10th exon of the gene [38]. The site of this insertion was verified by PCR followed by sequencing and the allele was designated *svr3-2 *(Figure 3A); homozygous *svr3-2 *plants resemble *svr3-1 *plants (Figure 3B). Semi-quantitative RT-PCR shows that the transcript of At5g13650 was not detectable in *svr3-2 *seedlings (Figure 3C). We also obtained *svr3-2 var2-5 *double mutants, and found that *var2 *variegation is suppressed in these plants (Figure 3B). The *svr3-2 var2-5 *double mutants are also paler and smaller than *svr3-2 *single mutant and wild-type plants. The genetic interaction between *svr3-2 *and *var2-5 *resembles those between *svr3-1 *and *var2-5*, again suggesting that the interaction between these alleles is complex. The acquisition of this second allele of *svr3 *supports our conclusion that At5g13650 is *SVR3*.

### *SVR3 *encodes a putative chloroplast TypA translation elongation factor

**Figure 4:**
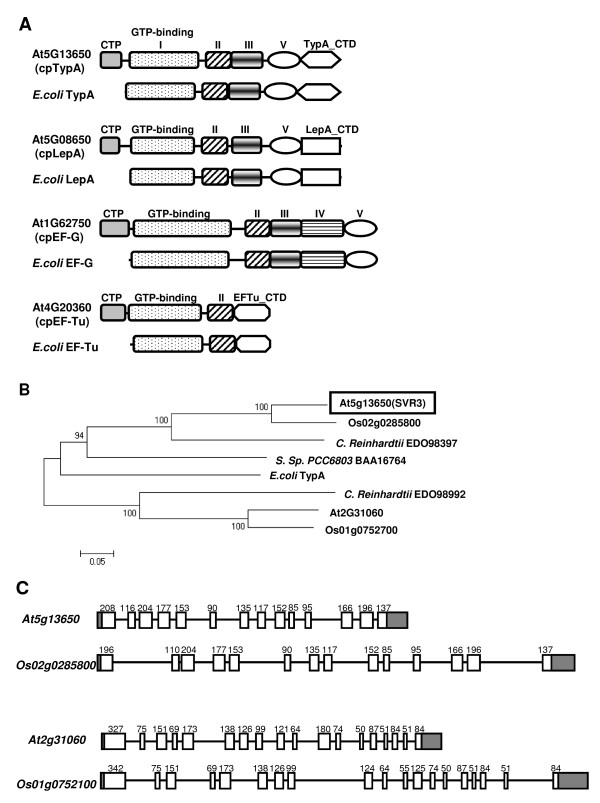
**Bioinformatics analysis of SVR3**. **(A) **Domain architecture of translation elongation factor GTPases. Chloroplast transit peptides (CTP) were predicted by TargetP [42]. Conserved domains were identified using InterProScan http://www.ebi.ac.uk/Tools/InterProScan/[82]. Arabidopsis protein sequences were obtained from TAIR http://www.Arabidopsis.org. *E. coli *protein sequences were obtained from uniprot.org (Accession numbers: EF-Tu, P0A6N1; EF-G, P0A6M8; LepA, P60785; TypA, P32132). **(B) **Phylogenetic tree of TypA homologs from Arabidopsis, rice, *Chlamydomonas reinhardtii*, *Synechocystis sp*. PCC6803 and *E. coli*. Full length protein sequences were obtained from the National Center for Biotechnology Information (NCBI). Gene ID or Genbank accession number is listed in the figure. MEGA4 software [83] was used for sequence analysis and phylogenetic tree construction. **(C) **Conservation of *TypA-like *gene structures in Arabidopsis and rice. Gene models were constructed based on annotation of the Arabidopsis and rice genomes. Boxes represent exons and lines represent introns. 5' and 3' untranslated regions (UTRs) are shaded. Numbers above each box refer to the number of nucleotides of each exon excluding the UTRs.

The translation product of *SVR3 *is predicted to contain 676 amino acids (~74.4 kDa), and it bears high similarity to the *E. coli *translation factor TypA (also known as BipA or YihK) (43% amino acid sequence identity, Additional file 1, Figure S2). TypA belongs to the family of translation elongation factor GTPases that include EF-G, EF-Tu and LepA [35]. A comparison of the domain structures of TypA, LepA, EF-G, and EF-Tu from *E. coli *and their putative chloroplast counterparts in Arabidopsis is shown in Figure 4A. It is notable that, with the exception of a putative chloroplast transit peptide (CTP) at the N-terminus of the chloroplast-targeted gene products in Arabidopsis (Figure 4A; Additional file 1, Figure S2), the domains of each factor are highly conserved between the two species. In addition, the four factors have many domains in common. A GTP binding domain (Domain I) is present in all factors, while TypA, LepA and EF-G share an additional three domains (Domains II, III and V) [39,40]. EF-G contains a unique domain IV whereas LepA and TypA each have a unique C-terminal domain (CTD). The overall domain structure of TypA is most similar to LepA, which promotes back translocation of peptidyl-tRNA from P site to A site and deacylated tRNA from E site to P site, the reverse reaction that is promoted by EF-G [41].

The TypA translation factor is widely but not universally found in prokaryotes and eukaryotes [35]. A phylogenetic analysis was performed to investigate the relationship of TypA homologs in representative photosynthetic organisms (Figure 4B). Only one copy of the *TypA *gene is found in *E. coli *and the photosynthetic cyanobacterium *Synechocystis sp*. PCC6803. However, two *TypA*-like genes are present in *Chlamydomonas reinhardtii*, rice and Arabidopsis. The products of these genes fall into two distinct clades. The corresponding Arabidopsis and rice genes in each clade having extraordinarily conserved exon structures in terms of exon numbers and sizes, suggesting a common evolutionary ancestor and maybe related functions (Figure 4C). Interestingly, SVR3/At5g13650 is more closely related to *E. coli *TypA than to the second Arabidopsis TypA-like protein, At2g31060 (Figure 4B).

### Plastid localization of SVR3

**Figure 5:**
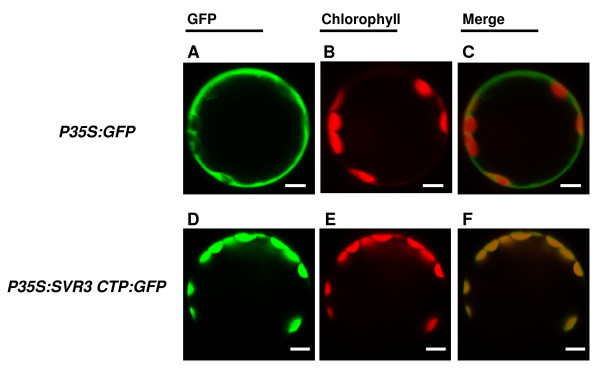
**Chloroplast localization of SVR3**. Representative wild-type Arabidopsis leaf protoplasts transiently expressing the control GFP vector (**[A]**-**[C]**) or the *P35S:SVR3 CTP:GFP *vector (**[D]**-**[F]**). Green fluorescence signals from GFP (**[A] **and **[D]**) and chlorophyll autofluorescence (**[B] **and **[E]**) were monitored by confocal microscopy. **(C) **and **(F) **are merged images from **(A) **&**(B) **and **(D) **&**(E)**, respectively. Bar represents 5 μm.

Compared to *E. coli *TypA, SVR3 has a long N-terminal extension (Additional file 1, Figure S2) that is predicted to be a chloroplast transit peptide (CTP) of 57 amino acids [42] and SVR3 has been identified as a chloroplast protein in several chloroplast proteome studies [43-46]. To confirm the chloroplast location of SVR3, a construct was generated that contained the SVR3 N-terminal region (1-64aa) fused with eGFP under the control of the CaMV 35 S promoter (designated *P35S:SVR3CTP:GFP*), and the construct was transiently expressed in wild-type Arabidopsis leaf protoplasts. A control construct contained only eGFP (designated *P35S:GFP*). Figure 5 shows that the green fluorescence signal from the control construct is present in the cytosol (Figure 5A-C), but that the green fluorescence from *P35S:SVR3 CTP:GFP *colocalized exclusively with chlorophyll autofluorescence (Figure 5D-F). These results indicate that the transit peptide of SVR3 is sufficient to direct a protein into the chloroplast, suggesting that SVR3 is a chloroplast protein.

### Chloroplast rRNA processing defects in *TAG-11*

**Figure 6:**
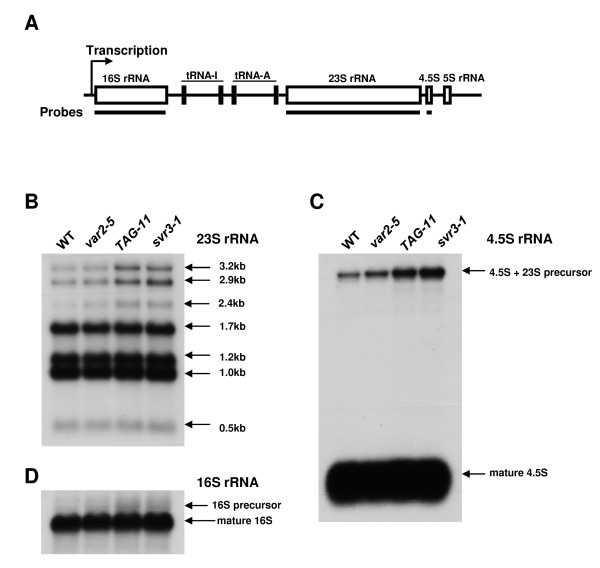
**Accumulation patterns of chloroplast rRNA transcripts at 22°C**. **(A) **Structure of *rrn *operon. Solid lines under each rRNA gene represent the probe used for Northern blot analysis in **(B)**-**(D)**. **(B)**-**(D) **Northern blots of 23 S **(B)**, 4.5 S **(C)**, and 16 S **(D) **rRNAs. Total leaf RNAs were extracted from three-week-old plants grown under the same conditions as shown in Figure 1A. Equal amounts of RNA (3 μg) were loaded onto each lane of the gel. After electrophoresis and transfer, nylon membranes were hybridized with ^32^P labeled rRNA gene-specific probes as indicated in **(A)**. The gel loading controls are shown in Additional file 1, Figure S5.

Chloroplast rRNA genes (23 S, 16 S, 4.5 S and 5S) are arranged in single transcription units, *rrn *operons in the chloroplast genome (Figure 6A). After transcription, a series of endonuclease cleavage and exonuclease trimming events are required for the maturation of each rRNA species [47]. Because chloroplast rRNA processing defects have been observed in several *var2 *suppressor lines [32,34], we wanted to address this question in the *svr3 *and *TAG-11 *plants. For these analyses, total cellular RNAs were extracted from wild-type, *var2-5*, *svr3-1*, and *TAG-11 *(*var2-5 svr3-1*) and Northern blot analyses were carried out using rRNA gene-specific probes. Accumulation patterns of the 23 S rRNA, 16 S rRNA and 4.5 S rRNA species reveal that their processing is not drastically altered in either *TAG-11 *or *svr3-1 *(Figures 6B, C and 6D respectively). However, higher molecular weight precursor forms of all three accumulate to somewhat higher levels in *TAG-11 *and *svr3-1 *compared to wild-type or *var2-5*. Considered together, our data suggest that *svr3 *has a small but measurable impact on chloroplast rRNA processing.

### Accumulation of chloroplast proteins in *TAG-11*

**Figure 7:**
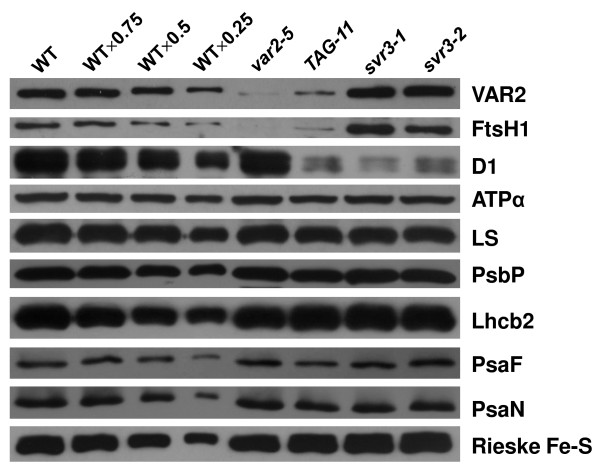
**Accumulation of chloroplast proteins at 22°C**. Total leaf proteins were extracted from two-week-old seedlings of wild-type, *var2-5*, *TAG-11 *(*var2-5 svr3-1*), *svr3-1 *and *svr3-2 *grown under the same conditions as in Figure 1A. A dilution series of the wild-type samples were loaded. Other samples were standardized to equal amounts of fresh tissue. Immunoblots were performed using polyclonal antibodies against chloroplast proteins of representative complexes: FtsH complex (VAR2, AtFtsH1), PSII (D1, PsbP), PSI (PsaF, PsaN), ATP synthase (ATPα), Rubisco (large subunit [LS]), Light harvesting complex (Lhcb2) and Cytochrome b_6_f (Rieske Fe-S). Plastid encoded proteins are D1, ATPα and Rubisco large subunit (LS). Nuclear encoded proteins are VAR2, AtFtH1, PsbP, PsaF, PsaN, Lhcb2 and Rieske Fe-S.

Though we did not find major defects in chloroplast rRNA processing in *svr3 *mutants, we were interested in determining whether the loss of SVR3 affects the accumulation of chloroplast proteins, given that SVR3 is a putative chloroplast translation elongation factor. To this end, we carried out immunoblot analysis on total leaf proteins from two-week-old seedlings (wild-type, *var2-5*, *TAG-11*, *svr3-1 *and *svr3-2*) using antibodies against representative chloroplast proteins encoded by both the nuclear and plastid genomes (Figure 7). We found that the levels of the VAR2 and AtFtsH1 subunits of thylakoid membrane FtsH complexes are considerably reduced in amount in *var2-5 *and *TAG-11*. This is as anticipated since reductions in the A pair of AtFtsH subunits are matched by reductions in the B pair, and vice versa, likely via post-translational turnover [29]. The coordinate reductions in VAR2 (Type B) and AtFtsH1 (Type A) [19] further suggest that suppression of variegation in *TAG-11 *is not due to enhanced expression/stability of FtsH subunit proteins. Figure 7 shows that the levels of most other proteins we examined do not appear to be significantly perturbed in the various mutant lines, with the exception of the D1 protein of PSII, which surprisingly was drastically reduced in amount in *TAG-11 *and the *svr3 *single mutants. In these plants, D1 is present at far less than 25% of the wild-type amount. This suggests that SVR3 is important for D1 accumulation.

### SVR3 is required for normal chloroplast biogenesis under chilling stress

**Figure 8:**
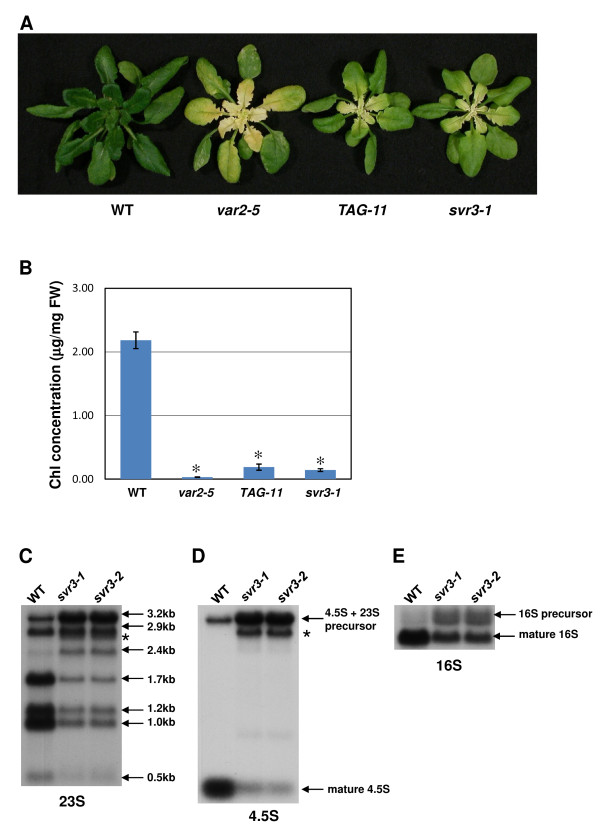
**Chilling sensitivity of *svr3***. **(A) **Phenotypes of seven-week-old wild-type, *var2-5*, *TAG-11 *and *svr3-1*. Plants were germinated and maintained at 22°C for three weeks before subjected to the chilling treatment at 8°C for four weeks. **(B) **Chlorophyll accumulation in the emerging yellow leaf tissues of the mutant and emerging green leaf tissues of wild-type (*: p < 0.01). **(C)**-**(E) **Accumulation patterns of chloroplast rRNA transcripts at 8°C. Northern blots of 23 S **(C)**, 4.5 S **(D)**, and 16 S **(E) **rRNAs were carried out with total RNA samples extracted from the same tissues as in **(B)**. Northern blot analysis with indicated probes was performed as in Figure 6. Gel loading controls are shown in Additional file 1, Figure S5.

Because compromised chloroplast translation often leads to a chilling sensitive phenotype (e.g., [48,49]), we were prompted to assess whether chloroplast biogenesis at low temperature is affected in *svr3*; i.e. whether TypA might be involved in the response to chilling stress. Figure 8A shows the phenotypes of seven-week-old wild-type, *var2-5*, *TAG-11 *and *svr3-1 *(grown at 22°C for three weeks and then transferred to 8°C for four weeks). At 8°C, wild-type plants maintained their ability to produce green leaves. By contrast, the emerging leaves in all mutant lines have a pronounced chlorosis phenotype due to decreased chlorophyll accumulation (Figure 8B), suggesting a compromised chloroplast development. The chilling sensitive phenotype of *svr3-1 *was further confirmed in *svr3-2 *and *svr3-1*/*svr3-2 *plants, indicating that they are allelic (Additional file 1, Figure S3).

To investigate whether the chlorosis phenotype of *svr3 *is due to perturbed chloroplast translation under chilling stress, Northern blot analysis were used to profile the accumulation of several chloroplast rRNA species in samples of total cellular RNA from yellow leaf tissues that developed at 8°C (Figure 8C-E). RNA samples from emerging wild-type leaves (green) served as control. Inspection of ethidium bromide-stained RNA gel shows that chloroplast mature rRNA species are greatly reduced in abundance in *svr3-1 *and *svr3-2 *but not in wild-type when grown at 8°C (Additional file 1, Figure S5D-F). The accumulation pattern of 23 S rRNA is shown in Figure 8C. In agreement with the stained RNA gel, the mature forms of 23 S rRNAs (1.2 kb, 1.0 kb and 0.5 kb) are greatly reduced in amount in both *svr3 *alleles while the precursor forms (3.2 kb, 2.9 kb and 2.4 kb) have an increased abundance. In addition, close examination of the blot revealed that there is a shadowy band (indicated by the asterisk) below the 2.9 kb processing intermediate in *svr3-1 *and *svr3-2 *but not in wild-type, suggesting there might be an additional abnormal processing site of 23 S rRNA in *svr3 *mutants. This was confirmed by Northern blot analyses using 4.5 S rRNA as a probe: in wild-type, only two bands, the 3.2 kb 23S-4.5 S dicistronic precursor and the mature form of 4.5 S rRNA, can be detected, whereas an additional band of ~2.9 kb is present in *svr3-1 *and *svr3-2 *(Figure 8D). This indicates that 23 S rRNA is abnormally processed closer to its 5'-end in the mutants and this band likely is the shadowy band we observed with 23 S rRNA probe. Figure 8E shows the results of Northern blot analysis using the16 S rRNA probe. As with 23 S rRNA and 4.5 S rRNA, the precursor form of 16 S rRNA accumulated to a much higher level in *svr3 *mutants while there was a reduction in the mature form. Our results suggest that SVR3 is required for normal chloroplast rRNA processing at 8°C.

**Figure 9:**
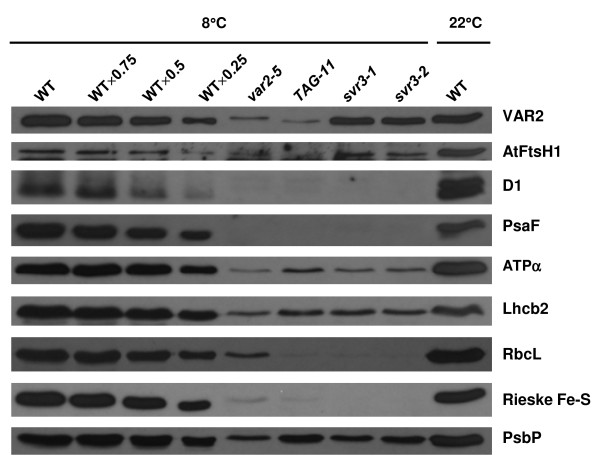
**Accumulation of chloroplast proteins at 8°C**. Total protein was extracted from same tissues as in **Figure 8B **and immnunoblot analysis was carried out as in **Figure 7**.

We next carried out immunoblot analysis to determine the levels of representative nuclear and plastid encoded proteins in leaf tissues from the mutant and wild-type plants that developed at 8°C (Figure 9). These analyses revealed that the levels of most proteins are not markedly affected by chilling temperatures in the wild-type, the exceptions being D1 and AtFtsH1, which were reduced about 50% at 8°C versus 22°C. Figure 9 further reveals that there are dramatic reductions in all proteins in the mutant lines (*var2-5*, *svr3-1 *and *TAG-11*) compared to wild-type, but in particular in the amounts of D1, PsaF, LS, and the Rieske Fe-S protein, which are barely detectable at the chilling temperature. This indicates that chloroplast-encoded proteins are not preferentially affected by the 8°C treatment. It is possible that SVR3 affects the accumulation of chloroplast DNA-encoded proteins at 8°C via disrupting chloroplast translation, and that the failure to synthesize chloroplast-encoded subunits of photosynthetic complexes might cause the turnover of unassembled nuclear-encoded subunits of the same complexes.

### Genetic interaction between *svr3 *and *svr7*

Distinct rRNA processing defects have been observed in a number of different *svr *mutant lines [34], suggesting that this process requires various factors. One of these mutants is *svr7*. The *svr7 *mutant, identified in our *var2 *suppressor screen, has a pale green phenotype similar to *svr3*. It is impaired in a chloroplast PPR protein containing a SMR domain at its C-terminus [34]. PPR proteins are RNA-binding proteins that are involved in the post-transcriptional regulation of organelle gene expression [50].

**Figure 10:**
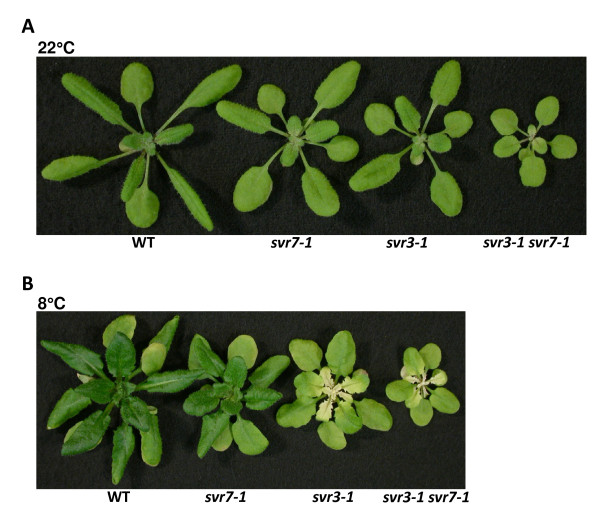
**Genetic interaction between *svr3 *and *svr7***. **(A) **Phenotypes of wild-type, *svr7-1*, *svr3-1 *and *svr3-1 svr7-1 *double mutant plants grown at 22°C for three weeks. **(B) **Phenotypes of wild-type, *svr7-1*, *svr3-1 *and *svr3-1 svr7-1 *double mutant plants grown at 22°C for three weeks followed by four weeks of growth at 8°C.

As an initial step to investigate the factors that are required in chloroplast rRNA processing, we undertook a genetic approach and generated double mutants between *svr3 *and *svr7*. The genotype of the *svr3-1 svr7-1 *double mutant was confirmed by a PCR assay (Addition file 1, Figure S4). The *svr3-1 *mutant allele contains a T-DNA insertion, so PCR will fail to amplify the fragment bearing the T-DNA insert from homozygous *svr3-1 *plant genomic DNA (Figure 2; Addition file 1, Figure S4). The *svr7-1 *allele contains 10 bp deletion in the *SVR7 *gene, and the size difference between the wild-type *SVR7 *allele and the *svr7-1 *allele can be distinguished by PCR (Addition file 1, Figure S4; [34]). The phenotype of the *svr3-1 svr7-1 *double mutants was examined at 22°C (Figure 10A) and 8°C (Figure 10B). The double mutant is much smaller and yellower than either of the single mutants at 22°C. At 8°C, even though the *svr7-1 *single mutant is resistant to cold treatment, the *svr3-1 svr7-1 *double mutant is susceptible to it inasmuch that the double mutant shows a chlorosis phenotype similar to that of the *svr3-1 *single mutant (Figure 10B). Double mutant analysis suggests SVR3 and SVR7 act in different pathways in promoting chloroplast development.

## Discussion

### Possible functions of SVR3

In this report, we found that loss of SVR3, a putative chloroplast TypA translation elongation GTPase, suppresses variegation mediated by *var2*, and that SVR3 is essential for plants' ability to develop functional chloroplasts under chilling stress (8°C), but not at normal temperature (22°C). The TypA translation factor is widely conserved but not universally present in all prokaryotes [35], suggesting that it is probably not an essential translation factor. This is consistent with our data that SVR3 is not essential for plant growth and chloroplast biogenesis at normal growth temperature. The subtle phenotype of *svr3 *at normal temperature and the fact that it is expressed at this temperature suggest that it probably plays a minor role in chloroplast translation at 22°C. At low temperature, however, SVR3 may become more intimately involved in chloroplast translation and the lack of SVR3 leads to more pronounced growth defects. Nevertheless, an alternative hypothesis is that SVR3/TypA might be a general stress related protein in plants.

The function of TypA has been studied extensively in prokaryotic systems and it is involved in a diverse array of processes including response to bactericidal proteins [51,52], virulence [53,54], capsule formation [55], symbiosis [56] and growth under adverse conditions such as low pH, and the presence of SDS [56]. In *Salmonella enterica*, TypA is able to compete with EF-G in ribosome binding, and the GTPase activity of TypA is stimulated in the presence of ribosomes [40]. It is notable that TypA is required for several bacteria species to grown at low temperatures [57-60], which is consistent with our findings that SVR3 is required for chloroplast biogenesis at low temperature. However, the exact role of TypA or SVR3 at low temperature is still not clear.

In plants, TypA-like proteins have been linked to the development of male reproductive organs [61,62]. The expression of *TypA *in *Suaeda salsa*, a salt resistant plant species, is responsive to oxidative stresses and ectopic overexpression of this gene resulted in increased oxidative tolerance in tobacco plants[63]. However, it is not clear whether TypA directly regulates these cellular processes, or alternatively, whether it primarily regulates ribosome function under various abiotic stresses, and all other processes are affected secondarily.

Translation elongation factors EF-Tu, EF-G, LepA and TypA share a similar arrangement of functional domains, especially the latter three, which share domains I, II, III and V and each also contains a unique domain (Figure 4A). Crystal structures of LepA and EF-G revealed highly similar three-dimensional structures [39,64]. Domains I and II are well conserved and provide sites for interaction with the 50 S and 30 S subunits of the ribosome, while the remaining three domains mediate interactions between LepA, EF-G with the A site of the ribosome [39,64]. A high resolution TypA crystal structure is not yet available but based on the extraordinarily conserved domain arrangement between TypA and other two translation elongation factors, we can predict that SVR3/AtcpTypA interacts with chloroplast ribosomes in a manner similar to those of LepA and EF-G with bacterial ribosomes.

Despite the above discussed similarities between translation elongation factors, it is likely that each factor also has its own features since each factor contains a unique domain, which might mediate factor specific interactions with the ribosome and facilitate different roles in translation. In the case of SVR3/AtcpTypA, the C-terminal domain may play a crucial role in mediating specific interactions between TypA and the ribosome at chilling temperature by mediating specific translation events. For example, we observed a specific reduction of photosystem II reaction center D1 proteins, but not of other plastid genome encoded proteins, in *svr3 *mutants. This certainly raises the possibility that SVR3 is specifically required for D1 translation in the chloroplast.

Chlorosis is one common phenotype observed in chilling-injury due to various reasons [48]. Compromised chloroplast translation is often found in chilling-sensitive mutants. Early studies with maize mutants such as M-11 [65], *v16 *[66] and *hcf*7 [67], showed that these mutants not only display chlorosis but also have more severe defects in chloroplast ribosome assembly and/or translation while exposed to low temperature. In tobacco, a mutant lacking the non-essential plastid coded ribosomal protein L33 has defects recovering from chilling injury [49]. Chilling stress in tobacco has also been associated with the pausing and delay of chloroplast ribosomes during translation elongation of *psbA *mRNA which in turn results in reduced synthesis of D1 protein [68,69]. In Arabidopsis, a decreased level of plastid protein accumulation has been described in the *chilling sensitive1 *(*chs1*) mutant [70]. A second Arabidopsis mutant, *paleface1 *(*pfc1*), defines a gene encoding a homolog of yeast 18 S rRNA dimethylase (DIM1). The phenotype of *pfc1 *is similar to *svr3 *inasmuch as it is indistinguishable from wild-type at normal temperature but displays a chlorosis phenotype at chilling temperature. The source of this chilling sensitivity was traced to an adenosine modification in chloroplast 16 S rRNA, which was abolished in *pfc1*, providing direct evidence that chloroplast rRNA processing defects can cause plant chilling-sensitivity [48]. On the other hand, a perturbed chloroplast rRNA processing and/or translation does not necessarily lead to chilling sensitivity [34], suggesting that chilling sensitivity is induced by defect(s) of a specific aspect(s) of chloroplast translation, rather than to a general compromised translation.

It is important to note that SVR3, as a translation elongation factor, is not expected to be a basic protein component of the chloroplast ribosome *per se*. Rather we propose that SVR3 is a regulatory protein that plays a role in translating specific proteins and that is more crucial during stress conditions. It is thus interesting to note that SVR3 protein levels have been found to be elevated in several chloroplast mutant backgrounds, such as mutants of *ClpR2 *and *ClpR4 *protease genes, suggesting that SVR3 may be part of a response pathway that is activated under stress and some other conditions [71,72]. Although we do not know how the absence of a regulatory protein such as SVR3 leads to impaired processing of chloroplast rRNA, our data add another factor to the growing list of proteins that have been implicated in the processing of chloroplast rRNAs [32]. At this stage, we do not yet know why there is reduced chloroplast rRNA/ribosome accumulation in *svr3 *at chilling temperatures, nor why there is abnormal rRNA processing and whether these two events are linked. There are at least three possible scenarios. One is that SVR3 might bind to ribosomes directly during ribosome assembly at chilling temperature. This interaction might protect the 23 S rRNA from being processed by endo- and/or exo-nucleases. The abnormally processed 23 S rRNA would destabilize ribosomes and eventually prevent them from achieving the maximum translation efficiency, which could be critical during the early stages of chloroplast biogenesis under chilling stress. A second possibility is that, instead of affecting chloroplast ribosome biogenesis directly, SVR3 might be important for the robust translation of a factor(s) that is required for chilling tolerance during the transition from proplastids to chloroplasts, and that lack of this factor(s) could lead to the abnormal processing event. Another possible explanation is that the *svr3 *mutation slows down chloroplast translation at low temperature, which reduces the rate of ribosomal protein synthesis, and in turn slows down ribosome assembly and rRNA processing.

The dramatic rRNA processing defects and loss of chloroplast proteins at low growth temperatures in *svr3 *are not common phenomena observed in other *svr *mutants. For example, *svr7*, in which a chloroplast PPR protein is disrupted, is quite resistant to cold stress and shows similar chloroplast rRNA and proteins accumulation patterns under normal and cold growth conditions [34].

### Mechanism of *var2 *suppression in *TAG-11*

Previously, a number of studies have established a link between compromised chloroplast translation and suppression of *var2 *[31,32,34]. The identification of *SVR3*, which encodes a putative chloroplast TypA translation elongation factor, reinforces this notion. However, one distinctive phenotype of *TAG-11 *is that the genetic interaction between *var2-5 *and *svr3 *is not epistatic as seen in other suppressor lines [30-32] in that the single *svr3 *mutant resembles many other suppressor single mutants and has a slightly pale green leaf color, but the double mutant suppressor line *TAG-11 *is smaller than *svr3 *single mutants and displays some variegation at later development stages. This is true for both alleles of *svr3*, indicating that it is specific for the *SVR3 *locus, rather than due to independent mutations in the *svr3-1 *and *svr3-2 *backgrounds. The incomplete suppression of variegation in *TAG-11 *raises the question about the complexity of the interaction between chloroplast translation and VAR2 function.

Though the exact role of VAR2 in chloroplast translation is unclear, both ours and other's genetic data have clearly established a link between VAR2 and chloroplast translation. The notion that VAR2 may be directly involved in chloroplast translation is not far-fetched and in fact is in agreement with findings in mitochondria, where an FtsH-like protease m-AAA, consisting of two homologous subunits YTA10 and YTA12, has been shown to be involved in the degradation of a number of mitochondrial inner membrane proteins [73]. In a landmark finding by Thomas Langer's group, the authors identified proteins that interact with the m-AAA complex [74]. Surprisingly, these include MrpL32, a ribosomal protein of the 50 S subunit of the mitochondrial 70 S ribosome encoded by the nuclear genome. The authors were able to demonstrate that m-AAA is responsible for processing of the MrpL32 precursor after it is translocated into the mitochondria but prior to its integration into the 70 S ribosome. Furthermore, many defects of *yta10 *and *yta12 *mutants can be rescued by simply providing the mature form of MrpL32 in the mitochondria, indicating that the failure to properly process MrpL32 is the underlying cause of *yta10 *and *yta12 *mutant phenotypes [74].

Currently there are no data suggesting similar direct interaction between VAR2 and its homologues with chloroplast ribosome. Early findings with chloroplast ribosomes have established that there are at least two sub-groups of chloroplast ribosomes: the stromal "free" ribosomes and the thylakoid-bound ribosomes [75,76]. On the other hand, FtsH complex containing VAR2 is situated in the thylakoid membrane. Thus it is conceivable that there might be functional relationships between these two complexes, particularly so considering the strong genetic link that has been established.

## Conclusions

In this report, we demonstrated that the disruption of *SVR3*, encoding a putative chloroplast TypA-type translation elongation factor, is the cause for the suppression of *var2*-mediated leaf variegation in *TAG-11 *suppressor line. *svr3 *mutations do not lead to major defects under normal growth temperature (22°C). However, at low temperature (8°C), the loss of SVR3 leads to major chloroplast rRNA processing defects and reduced chloroplast protein accumulations. This work identified a new *var2 *suppressor locus, reinforced the genetic link between VAR2 and chloroplast translation and also revealed a novel role for SVR3 in plant's responses to chilling stress.

## Methods

### Plant growth and maintenance

All *Arabidopsis thaliana *plants were maintained at 22°C under continuous illumination with a light intensity of ~100 μmol·m^-2^s^-1^. For the chilling treatment, plants were germinated and grown at 22°C for three weeks and then transferred to 8°C for another four weeks under the same illumination conditions. The *svr3-1 *single mutant was derived from *var2-5 *suppressor line *TAG-11 *while the *svr3-2 *single mutant was identified from the SAIL T-DNA insertion mutant library under the designation CS871763 [38]. The *svr7-1 *single mutant used in this study is derived from the *var2 *suppressor line *004-003 *[34]. All Arabidopsis mutants used in this study are generated in the Columbia ecotype background.

### Chlorophyll Measurements

Two-week-old seedlings were harvested, weighed and frozen in liquid nitrogen. Plant tissues were ground in liquid nitrogen and chlorophyll pigments were extracted using 95% ethanol with gentle shaking at 4°C overnight. Samples were then centrifuged at 14,000 g for 10 minutes at 4°C. The supernatants were diluted and used for light absorbance measurements at 664 nm and 649 nm. Chlorophyll content and chlorophyll a/b ratios were calculated according to [77].

### Map-based cloning of *SVR3*

Map-based cloning was performed according to [37]. In brief, suppressor line *TAG-11 *(*var2-5 svr3-1*) was crossed with Landsberg *erecta *to generate an F2 mapping population. The suppressor gene in *TAG-11 *was first mapped to a region adjacent to SSLP marker *nga151 *on chromosome 5 by bulked segregant analysis using pooled DNA from 100 F2 plants [78,79]. Additional molecular markers were designed based on Indel or SNP polymorphisms between Landsberg *erecta *and Columbia ecotypes [37] (Additional file 1, Table S1) to fine map the gene to a ~123 kb interval using a mapping population of 570 F2 plants (1140 chromosomes). PCR and RT-PCR primers that were used to confirm the T-DNA insertion site are listed in Additional file 1, Table S1.

### Plasmid construction and transient expression in protoplasts

A vector pTF486 (designated *P35S:GFP*) containing the open reading frame of *eGFP *driven by the CaMV 35 S promoter was used as a control construct [32]. The N-terminal region (1-64aa) of SVR3 encompassing the predicted chloroplast transit peptide was amplified using primers 13650GFPF and 13650GFPR (Additional file 1, Table S1) using *pfu *Turbo DNA polymerase (Stratagene, CA, USA). The PCR product was then cloned into the *BamH*I and *Nco*I sites of pTF486. The resulting construct was designated *P35S:SVR3 CTP:GFP*. Both *P35S:GFP *and *P35S:SVR3CTP:GFP *were introduced into wild-type Arabidopsis leaf protoplasts and transient GFP expression was observed [32,80]. The fluorescent signals of GFP and chlorophyll autofluorescence were monitored by confocal microscopy (Leica TCS NT) using a FITC-TRITC filter combination.

### Phylogenetic and gene structure analysis

Full-length protein sequences of SVR3/TypA homologs were obtained from the National Center for Biotechnology Information (NCBI) Genbank. The alignment of the sequences and the construction of the phylogenetic tree were performed as described in [32]. Gene structures of Arabidopsis and rice *TypA *homologs were constructed based on the annotation of the Arabidopsis genome from TAIR http://www.arabidopsis.org and rice genome from NCBI Genbank.

### Protein analysis

Total leaf proteins were isolated as previously described [29]. In brief, two-week-old seedlings were harvested and weighed, then ground in liquid nitrogen in 2 × SDS-PAGE sample buffer (0.125 M Tris, pH6.8, 4% SDS, 20% glycerol, 2% β-mercaptoethanol and 0.02% bromophenol blue) and centrifuged at 14,000 g for ten minutes. The supernatants were resolved via 12% SDS-PAGE, and the proteins were transferred onto nitrocellulose membranes (Immobilon-NC, Millipore, USA). Polyclonal antibodies described in [32] were used in the immunoblots. Proteins were detected using the SuperSignal West Pico chemiluminescence kit (Pierce, USA).

### Manipulation of nucleic acids

The CTAB method was used to extract Arabidopsis leaf DNA [81], and the Trizol RNA reagent (Invitrogen, CA, USA) was used to extract total leaf RNA. RNA gel analysis and Northern blots were performed as described in [32]. RT-PCR was performed according to [29]. Primers used for generation of probes used in Northern blots, RT-PCR of *ACTIN2*, and internal PCR control were described in [32]. Other primers used in this study are listed in Additional file 1, Table S1.

### Generation of *svr3 svr7 *double mutants

The *svr3-1 *single mutant was crossed with *svr7-1 *single mutant. The genotype of *SVR3 *and *SVR7 *loci in F2 progeny derived from the cross was determined by PCR analysis: PCR primers 13650F1 and 13650R1-1 was used to genotype *SVR3 *locus; PCR primers 004-003F and 004-003R were used to determine the genotype of the *SVR7 *locus.

### Accession numbers

SVR3/At5g13650: NP_851035; At2g31060: NP_001031452; rice TypA1: NP_001046573; rice TypA2: NP_001044268; *Chlamydomonas reinhardii *EDO98397: XP_001700103; *C*. *reinhardii *EDO98992: XP_001699137; *Synechocystis sp*. *PCC6803 *BAA16764: NP_440084; *E. coli *TypA: YP_026274.

## Authors' contributions

XL performed phenotype analysis, genetic mapping and molecular work for Figures 1, 2, 3, 4, 5, 7, 9, 10 Additional file 1, Figures S2, S3 and Table S1, FY carried out molecular work in Figures 6, 8, Additional file 1, Figures S1 and S4. SRR and FY conceived, directed and wrote the manuscript. All authors read and approved the final manuscript.

## Supplementary Material

Additional file 1 **Supplemental Materials**. **Figure S1**. Co-segregation analysis of *TAG-11*. **Figure S2**. Alignment of *E.coli *TypA and AtcpTypA (SVR3) sequences. **Figure S3**. Cold phenotype of WT, *svr3-1*, *svr3-2 *and *svr3-1/svr3-2*. **Figure S4**. Genotyping of the *svr3-1 svr7-1 *double mutant. **Figure S5**. Loading control for northern blots. **Table S1**. Primers used in this study. Click here for file
