# Supplementary material for: A var2 leaf variegation suppressor locus, SUPPRESSOR OF VARIEGATION3, encodes a putative chloroplast translation elongation factor that is important for chloroplast development in the cold
Source: BMC Plant Biol. 2010 Dec 28;10:287. doi: 10.1186/1471-2229-10-287 (PMC3022910; doi:10.1186/1471-2229-10-287)
Supplement: Additional file 1 — Supplemental Materials. Figure S1. Co-segregation analysis of TAG-11. Figure S2. Alignment of E.coli TypA and AtcpTypA (SVR3) sequences. Figure S3. Cold phenotype of WT, svr3-1, svr3-2 and svr3-1/svr3-2. Figure S4. Genotyping of the svr3-1 svr7-1 double mutant. Figure S5. Loading control for northern blots. Table S1. Primers used in this study. [file 1471-2229-10-287-S1.PDF]

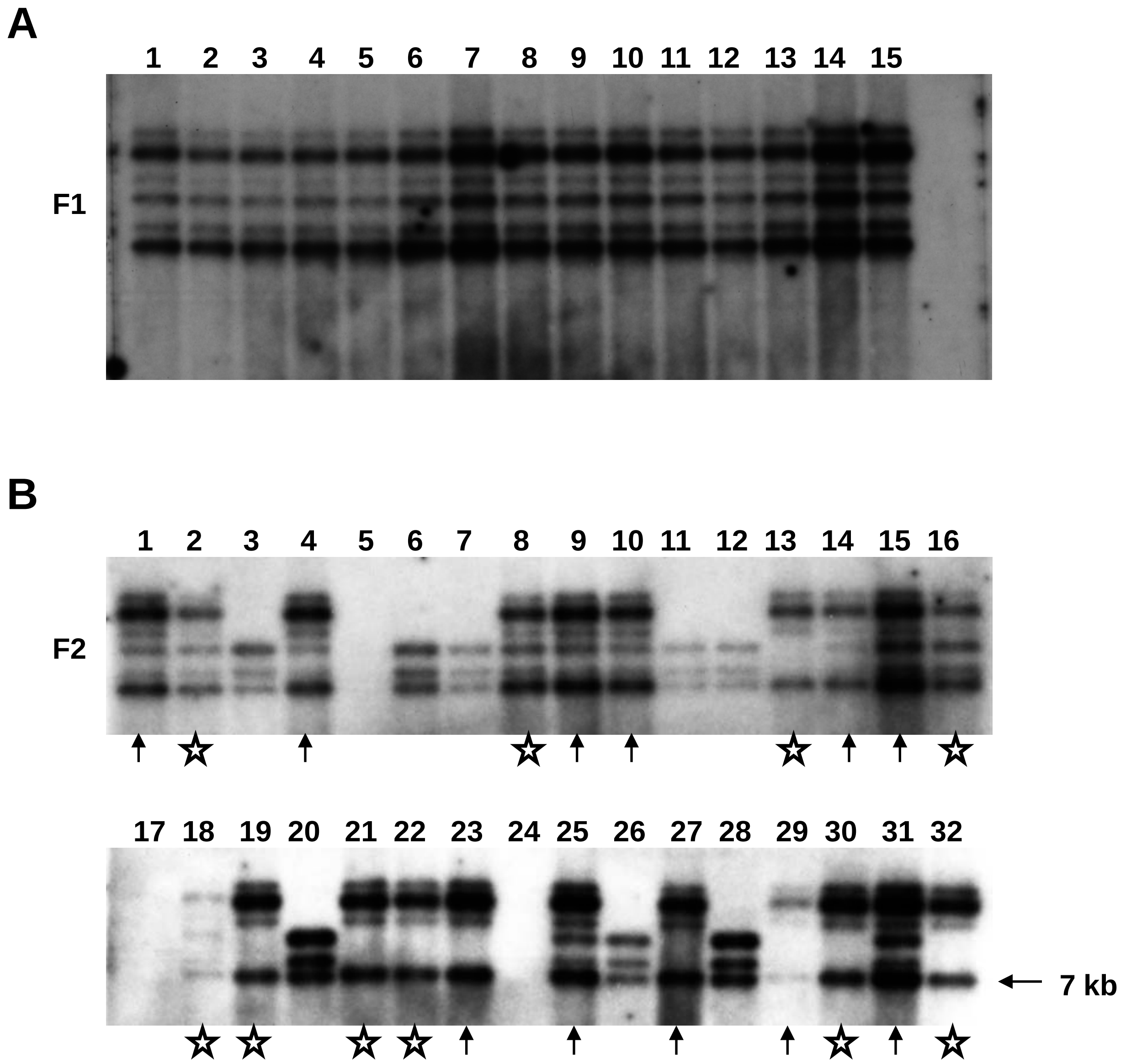

Additional file 1, Figure S1. Co-segregation analysis of *TAG-11*.

(A) Genomic DNAs from F1 plants of a cross between *TAG-11* and *var2-5* were extracted and digested with *Hind*III. The Southern blot was probed with *BAR* gene sequence (Yu et al., 2008). All F1 plants showed a *var2-5* phenotype and a complex banding pattern.

(B) Genomic DNAs from F2 plants derived from a cross of *TAG-11* and *var2-5* were extracted and digested with *Hind*III. The Southern blot was conducted as in **(A)**. In F2, the bands shown in **(A)** segregated into three patterns: the original complex pattern (as in **[A]**), a three-band pattern and a four-band pattern. In the 32 F2 plants examined by Southern analysis, all plants with a *TAG-11* phenotype had either the original or the four-band pattern (marked by arrows). F2 plants that had the original or the four-band pattern but a *var2-5* phenotype showed an approximately 3:1 *var2-5*: *TAG-11* ratio in the F3 generation (marked by stars). This result suggests that the four-band pattern, as shown by the “starred” plants, cosegregates with the *TAG-11* phenotype.

```
E.coliTypA      ------------------------------------------------------------
AtcpTypA        MELSLSTSSASPAVLRRQASPLLHKQQVLGVSFASALKPGGGALRFPSRRPLPRPITCSA 60

E.coliTypA      ------------------MIEKLRNIAIIAHVDHGKTTLVDKLLQQSGTFDSRAETQERV 42
AtcpTypA        SPSTAEPASVEVKKKQLDRRDNVRNIAIVAHVDHGKTTLVDSMLRQAKVFRDNQVMQERI 120
                                    :::*****:************.:*:*: .* ..   ***:

E.coliTypA      MDSNDLEKERGITILAKNTAIKWNDYRINIVDTPGHADFGGEVERVMSMVDSVLLVVDAF 102
AtcpTypA        MDSNDLERERGITILSKNTSITYKNTKVNIIDTPGHSDFGGEVERVLNMVDGVLLVVDSV 180
                *******:*******:***:*.::: ::**:*****:*********:.***.******:.

E.coliTypA      DGPMPQTRFVTKKAFAYGLKPIVVINKVDRPGARPDWVVDQVFDLFVNLDATDEQLDFPI 162
AtcpTypA        EGPMPQTRFVLKKALEFGHAVVVVVNKIDRPSARPEFVVNSTFELFIELNATDEQCDFQA 240
                :********* ***: :*   :**:**:***.***::**:..*:**::*:***** **

E.coliTypA      VYASALNGIAGLDHEDMAEDMTPLYQAIVDHVPAPDVDLDGPFQMQISQLDYNSYVGVIG 222
AtcpTypA        IYASGIKGKAGLSPDDLAEDLGPLFEAIIRCVPGPNIEKDGALQMLATNIEYDEHKGRIA 300
                :***.::* ***. :*:***: **::**:  **.*::: **.:**  ::::*:.: * *.

E.coliTypA      IGRIKRGKVKPNQQVTIIDSEGKTRNAKVGKVLGHLGLERIETDLAEAGDIVAITGLGEL 282
AtcpTypA        IGRLHAGVLRKGMDVRVCTSEDSCRFARVSELFVYEKFYRVPTDSVEAGDICAVCGIDNI 360
                ***:: * :: . :* :  **.. * *:*.::: :  : *: ** .***** *: *:.::

E.coliTypA      NISDTVCDTQNVEALPALSVDEPTVSMFFCVNTSPFCGKEGKFVTSRQILDRLNKELVHN 342
AtcpTypA        QIGETIADKVHGKPLPTIKVEEPTVKMSFSVNTSPFSGREGKYVTSRNLRDRLNRELERN 420
                :*.:*:.*. : :.**::.*:****.* *.******.*:***:****:: ****:** :*

E.coliTypA      VALRVEETEDADAFRVSGRGELHLSVLIENMRREGFELAVSRPKVIFREIDGRKQEPYEN 402
AtcpTypA        LAMKVEDGETADTFIVSGRGTLHITILIENMRREGYEFMVGPPKVINKRVNDKLLEPYEI 480
                :*::**: * **:* ***** **:::*********:*: *. **** :.::.:  ****

E.coliTypA      VTLDVEEQHQGSVMQALGERKGDLKNMNPDG-KGRVRLDYVIPSRGLIGFRSEFMTMTSG 461
AtcpTypA        ATVEVPEAHMGPVVELLGKRRGQMFDMQGVGSEGTTFLRYKIPTRGLLGLRNAILTASRG 540
                .*::* * * *.*:: **:*:*:: :*:  * :* . * * **:***:*:*. ::* : *

E.coliTypA      TGLLYSTFSHYDDVRPGEVGQRQNGVLISNGQGKAVAFALFGLQDRGKLFLGHGAEVYEG 521
AtcpTypA        TAILNTVFDSYG-PWAGDISTRDLGSLVAFEDGTSTSYALASAQERGQMFVGSGVDVYKG 599
                *.:* :.*. *.   .*::. *: * *::  :*.:.::** . *:**::*:* *.:**:*

E.coliTypA      QIIGIHSRSNDLTVNCLTGKKLTNMRASGTDEAVVLVPPIRMTLEQALEFIDDDELVEVT 581
AtcpTypA        QIVGIHQRPGDLGLNICKKKAATNIRSN-KDVTVILDTPLTYSLDDCIEYIEEDELVEVT 658
                **:***.*..** :*  . *  **:*:. .* :*:* .*:  :*::.:*:*::*******

E.coliTypA      PTSIRIRKRHLTENDRRRANRAPKDD 607
AtcpTypA        PSSIRMCKNQKMAKKGRQ-------- 676
                *:***: *.:   :. *:
```

Additional file 1, Figure S2. Alignment of *E.coli* TypA and AtcpTypA (SVR3) sequences.

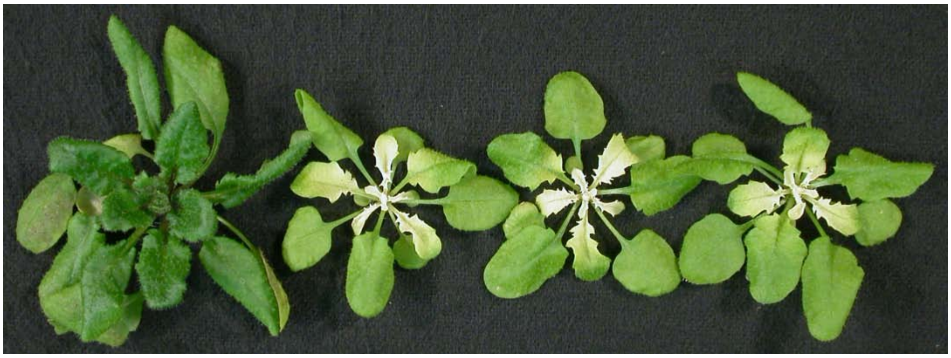

WT ***svr3-1*** ***svr3-2*** ***svr3-1/svr3-2***

Additional file 1, Figure S3. Cold phenotype of WT, *svr3-1*, *svr3-2* and *svr3-1/svr3-2*.

Plants were first grown at 22ºC for three weeks and then transferred to 8ºC for another four weeks before photographing.

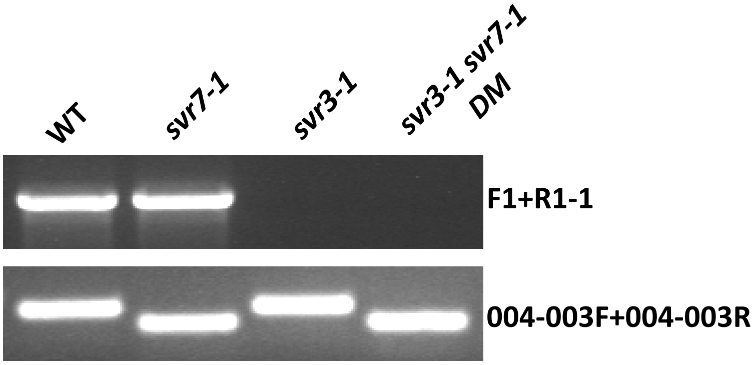

Additional file 1, Figure S4. Genotyping of the *svr3-1 svr7-1* double mutant. Top panel: The *SVR3* locus was genotyped by PCR using primers F1 and R1-1 flanking the T-DNA insertion site in *svr3-1*. Bottom panel: The *SVR7* locus was determined by PCR using primers 004-003F and 004-003R flanking the region containing the deletions in *svr7-1*. Primer sequences are listed in Table S1.

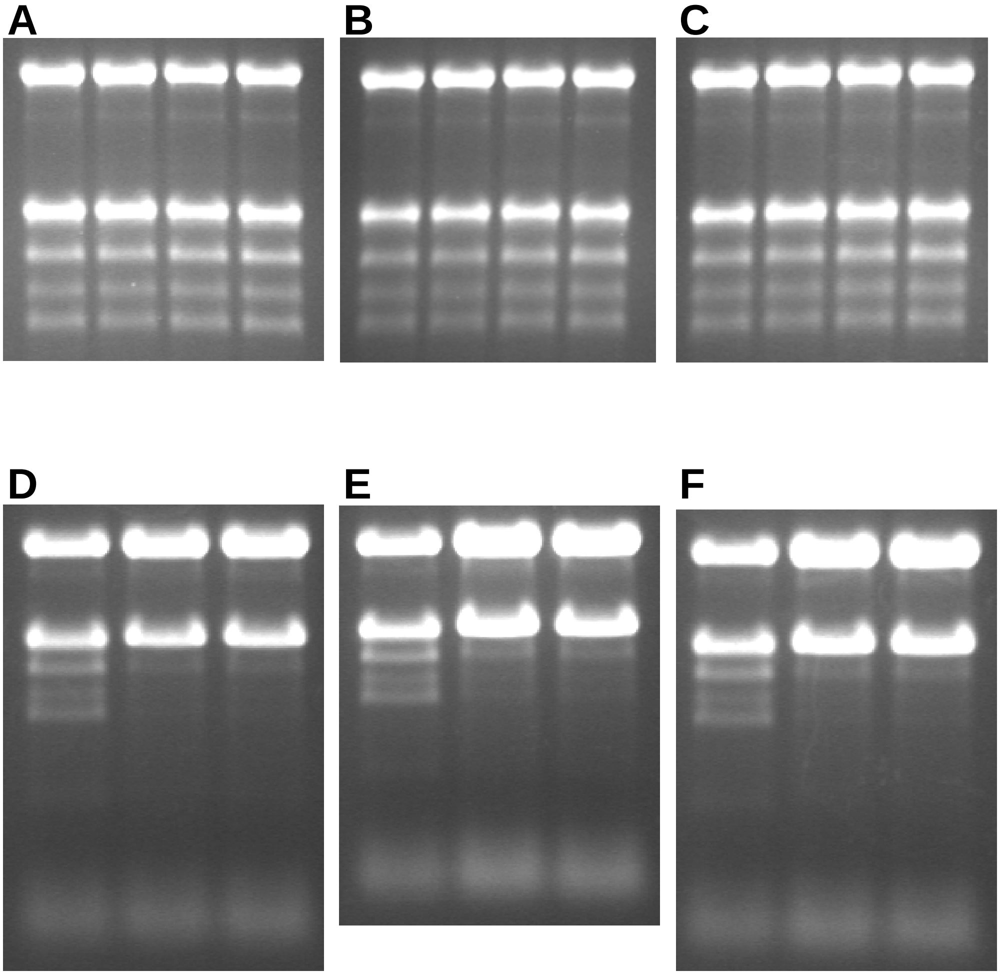

Additional file 1, Figure S5. Loading control for northern blots.

Ethidium bromide-stained RNA gels for the Northern blots in Figure 6B (panel A), Figure 6C (panel B), Figure 6D (panel C), Figure 8C (panel D), Figure 8D (panel E), and Figure 8E (panel F).

Additional file 1, Table S1. Primers used in this study.

Primer Name	Primer Sequence	Notes
13650F1C	5′-CAT TCT AGA TTC TCC CTT TTC TCT GTT TCG CG-3′	
13650R1C	5′-CAT TCT AGA CTT CAA TTT CCT CCA AAC CCA GC-3′	
13650F1	5′-TCG CTT AAT GAA ATC CTC GG-3′	
13650F1-1	5′-CGT GGT CGT AAA CAA GAT TG-3′	
13650F2	5′-GTG GCA TAG ACA ACA TTC AG-3′	
13650R1-1	5′-ATC CCA CTA GCA TAT ATC GC-3′	
13650R1	5′-GAT TGT AGG TAG AGG CTT CC-3′	
13650R3	5′-CAG TGT TAA GGA TAG CTG TC-3'	
13650GFPF	5′-CAT GGA TCC TTC TCC CTT TTC TCT GTT TCG CG-3′	
13650GFPR	5′-CAT GCC ATG GAA CCA CCA CCA CCA CCA CCA GTT GAT GGA GAG GCG GAG CA-3′	
T6I14#1F	5′-GGT TTC TTC TAT TAA GGA CC-3′	Indel col/Ler: 131bp/105bp
T6I14#1R	5′-TAA AAC ATC GTC GTC GGA TG-3′	
MXE10#1F	5′-TGC TTT TCT CCC TGG TAA TG-3′	Indel col/Ler: 110bp/98bp
MXE10#1R	5′-GTC TCA CTT GCT CAA GTT TG-3′	
MUA22#1F	5′-CTG GGT ATT GAT GGA CCA AG-3′	Indel col/Ler: 96bp/84bp
MUA22#1R	5′-TCT CGG CGA TGG CAA AAT CC-3′	
F18O22#1F	5′-GGT TAT ATC GAA AGC GAC TCT AG-3′	dCAPs w/XbaI
F18O22#1R	5′-GAC GAC ATT GGG AGA ATT AG-3′	

The Mg^{2+} concentration used for all PCR reactions is 1.5mM.
